# Peak exercise capacity estimated from incremental shuttle walking test in patients with COPD: a methodological study

**DOI:** 10.1186/1465-9921-7-127

**Published:** 2006-10-17

**Authors:** Ragnheiður Harpa Arnardóttir, Margareta Emtner, Hans Hedenström, Kjell Larsson, Gunnar Boman

**Affiliations:** 1 Department of Medical Sciences: Respiratory Medicine and Allergology, Uppsala University, Uppsala, Sweden; 2 Department of Medical Sciences: Clinical Physiology, Uppsala University, Uppsala, Sweden; 3 Lung and Allergy Research, National Institute of Environmental Medicine, Karolinska Institutet, Stockholm, Sweden

## Abstract

**Background:**

In patients with COPD, both laboratory exercise tests and field walking tests are used to assess physical performance. In laboratory tests, peak exercise capacity in watts (W peak) and/or peak oxygen uptake (VO_2 _peak) are assessed, whereas the performance on walking tests usually is expressed as distance walked. The aim of the study was to investigate the relationship between an incremental shuttle walking test (ISWT) and two laboratory cycle tests in order to assess whether W peak could be estimated from an ISWT.

**Methods:**

Ninety-three patients with moderate or severe COPD performed an ISWT, an incremental cycle test (ICT) to measure W peak and a semi-steady-state cycle test with breath-by-breath gas exchange analysis (CPET) to measure VO_2 _peak. Routine equations for conversion between cycle tests were used to estimate W peak from measured VO_2 _peak (CPET). Conversion equation for estimation of W peak from ISWT was found by univariate regression.

**Results:**

There was a significant correlation between W peak and distance walked on ISWT × body weight (r = 0.88, p < 0.0001). The agreement between W peak measured by ICT and estimated from ISWT was similar to the agreement between measured W peak (ICT) and W peak estimated from measured VO_2 _peak by CPET.

**Conclusion:**

Peak exercise capacity measured by an incremental cycle test could be estimated from an ISWT with similar accuracy as when estimated from peak oxygen uptake in patients with COPD.

## Background

Measurements of exercise capacity are important and widely used in rehabilitation of patients with chronic obstructive pulmonary disease (COPD). Exercise testing in COPD varies from maximal laboratory tests, requiring advanced technical equipment, to simple field tests. Maximal laboratory tests are mostly constructed to measure peak exercise capacity (W peak), and/or peak oxygen uptake (VO_2 _peak) whereas field tests have been considered to reflect functional capacity [1-3]. The incremental shuttle walking test (ISWT) is a field test which is similar to the laboratory tests as it is externally paced and progressive [4]. During ISWT there is a linear relationship between VO_2 _and walking speed, similar to the relationship between VO_2 _and work rate in incremental laboratory testing [5,6]. Singh et al found, when comparing incremental laboratory treadmill test and ISWT, that VO_2 _peak could be estimated from distance walked on ISWT [5].

Singh et al compared two different walking tests (treadmill and ISWT) and it is unclear whether such a strong relationship would also be found between ISWT and cycle performance. In laboratory testing, the treadmill test evokes slightly higher ratings of VO_2 _peak than the cycle tests [7,8], whereas different protocols of cycle tests usually result in similar VO_2 _peak but different W peak, depending on the slope of increased load during the test [9-11]. Body weight is an important contributor to the work load during walking, whereas it is of minor importance during cycling. Thus, the correlation between performance on timed walking tests and VO_2 _peak from a cycle test becomes stronger if distance walked is multiplied by body weight (distance × weight = work of walking at horizontal level) [12,13]. Recent findings indicate that metabolic and ventilatory responses to walking may differ from the responses to cycling in patients with COPD [8,14,15]. In pulmonary rehabilitation many exercise programmes are conducted on ergometer cycles. Target training intensity is often expressed as a percent of W peak, which in turn usually is defined as the highest work rate tolerated during an incremental cycle test with 1-minute steps or ramp increments. From a known VO_2 _peak it is possible to estimate W peak [16,17], and as VO_2 _peak can be estimated from an ISWT [5] it seems reasonable to assume that W peak could be estimated from ISWT through the estimated VO_2 _peak. However, using two conversion equations would make the results less reliable. Therefore, an estimation of W peak directly from the performance on ISWT would be preferable. This would be of clinical interest when expensive laboratory tests are not available.

The aim of the present study was to investigate whether W peak (assessed on a cycle ergometer) could be estimated from an ISWT in patients with moderate or severe COPD. For this purpose, comparisons of ISWT and two different cycle tests were made.

## Methods

### Material

Ninety-three subjects with moderate or severe COPD according to the British Thoracic Society [18] were consecutively invited to take part in the study when being referred for training to the Physiotherapy Unit of the Pulmonary Section at the Akademiska Hospital, Uppsala, Sweden during 2001–2004. All were smokers or ex-smokers. The study was approved by the Medical Ethics Committee of Uppsala University and all subjects gave informed consent.

Inclusion criteria were COPD with forced expiratory volume in one second (FEV_1_) < 60% of predicted value and FEV_1_/VC (vital capacity) < 0.7 after bronchodilatation [18]. Exclusion criteria were other diseases interfering with exercise such as ischemic coronary disease and musculo-skeletal problems.

### Testing

Lung function was measured with a Masterlab Trans spirometer, Masterlab Body Plethysmograph and Masterlab Transfer (Erich Jaeger AG, Würzburg, Germany) in accordance with the ATS guidelines [19]. Swedish reference values were used [20,21].

Incremental shuttle walking test (ISWT) was performed in a level corridor. Two cones were placed 9 m apart comprising a 10 m track as described by Singh et al [4]. Instructions to the subject and the pace of the test were played from a tape recorder. The test commenced at a speed of 30 m/min which then was increased by 10 m/min every minute. The subjects continued walking until they were not able to reach the next cone in time for the signal or got too exhausted to continue. The total distance walked was the main outcome of the test. Before and directly after walking, oxygen saturation (SpO_2_), heart rate, peak expiratory flow (PEF), perceived exertion (Borg RPE-scale) [22] and dyspnoea (Borg CR-10-scale) [23] were registered. The patients carried a pulse oximeter (Optovent Respons, Optovent, Linköping, Sweden) by a shoulder strap throughout the test. In 52 of the patients the test was identically repeated within a week.

Symptom-limited incremental cycle ergometer test (ICT) (Case 8000 Exercise Testing System, GE Medical Systems, Milwaukee, USA) with continuous ECG-registration was conducted to measure peak work load (W peak). The subjects started pedalling at 20 W and the load was then increased by 10 W every fulfilled minute until exhaustion. Heart rate, breathing frequency and SpO_2 _(Optovent Respons) were registered every minute during exercise. Systolic blood pressure, subjective ratings of perceived exertion and dyspnoea were recorded every other minute [22,23]. All variables were measured before and 1, 2, 4 and 10 minutes after exercise.

A semi-steady-state cardiopulmonary exercise test with breath-by-breath gas exchange analysis (CPET) was performed, according to routines at our clinic, after resting for at least 30 min after the incremental cycle test (longer if needed for all resting parameters to be stable at pre-exercise levels). Measurements of heart rate, SpO_2 _and ratings of perceived exertion and dyspnoea were made identically to the incremental cycle test. To enable measurement of VO_2 _peak, the subjects wore a mask with a turbine for gas exchange analysis (Oxycon Sigma, Jaeger, Germany). Additionally, VCO_2_(carbon dioxide)_, _minute ventilation (V_E_), respiratory quotient (RQ) and breathing frequency were measured with readings every 30 seconds. After recording steady-state measurements at rest (approximately 4 min of registration at rest) the patient started pedalling at 20 W. The load was kept constant until the ventilation and oxygen uptake reached a plateau, on average 3–4 min at each level, then the load was increased. To keep testing time within reasonable limits the load was increased until exhaustion by 5, 10, 20 or 30 W depending on the outcome of the first test (ICT).

The reason for discontinuing the cycle tests and the ISWT was stated at the end of each test.

The two different ergometer cycle tests were performed on the same day, but the lung function test and the ISWT were conducted on separate days. The three test days were separated by 1–3 resting days.

### Conversion equations

W peak was estimated from the measured VO_2 _peak on CPET by an equation derived from Åstrand [16]: (VO_2 _peak × 1000^-1^- 0.1517) × 0.0134^-1^. The equation from Singh et al for estimating VO_2 _from ISWT [5] was multiplied by body weight to express ml/min instead of ml/min/kg: (4.19 + 0.025 × walking distance) × body weight.

### Statistical analysis

Results were expressed as mean and standard deviation (SD) or 95% confidence interval (CI). For simple correlations Pearson's correlation coefficient was calculated. ANOVA and Student's *t*-test were calculated for paired comparisons, except for RPE and CR-10 scores where Friedman's ANOVA and Wilcoxon's signed rank test were used. Scatterplots as recommended by Bland and Altman were made for comparison of estimated and measured values. The level of significance was set at 5%.

## Results

All 93 subjects accepted to participate and were enrolled in the study, 71 with severe disease according to the British Thoracic Society guidelines [18]. There were no drop-outs. See Table 1 for characteristics.

**Table 1:** Characteristics of the patients. Values expressed as mean ± SD and (range), n = 93.

Gender, male/female	26/67		
Smoking habits, current/ex	23/70		
Age, years	64	± 7	(43–80)
Weight, kg	65.4	± 13.2	(42–101)
BMI, kg/m^2^	23.4	± 4.3	(14.5–34.9)
Packyears	36	± 21	(8–120)
VC, liters	2.6	± 0.9	(1.3–6.1)
VC, % predicted	67	± 16	(40–121)
FEV_1_, liters	0.9	± 0.3	(0.4–1.7)
FEV_1_, % predicted	32	± 11	(14–59)
MVV, liters/min	33.5	± 12.7	(15.0–66.7)
MVV, % predicted	46	± 12	(16–83)
DLCO, μmol/sec/kPa	57.2	± 22.4	(16.2–127)
DLCO, % predicted	50	± 18	(15–106)

BMI: body mass index; VC: vital capacity; FEV_1_: forced expiratory volume in one second; MVV: maximum voluntary ventilation; DLCO: carbon monoxide diffusion capacity.

### Test results

Distance walked on ISWT was 314 (291–336) m. End-exercise work load was 62 (57–68) W on the incremental cycle test (i.e. W peak) and 46 (41–50) W on the CPET. Measured peak VO_2 _on CPET was 973 (908–1038) ml/min. There was significantly lower peak heart rate, SpO_2_, ratings of perceived exertion and dyspnoea at the end of the walking test compared to the cycle tests (Table 2). The reported reasons for cessation were identical in the two cycle tests: 39% because of dyspnoea, 35% because of a combination of dyspnoea and exertion, 20% because of exertion and 6% because of leg fatigue. In the ISWT the reason for cessation was dyspnoea in 3%, a combination of dyspnoea and exertion in 12% and inability to increase or keep up the speed to reach the next cone in time in 85% of the cases.

**Table 2:** End-exercise values from the three tests: ISWT, ICT and CPET, n = 93.

	ISWT		ICT		CPET	
SpO_2, _%	87***	(86–88)	90	(89–91)	89^† ^	(88–91)
Heart rate, beats/min	115***	(111–118)	131	(127–138)	131	(124–134)
Perceived exertion, RPE	15.9***	(15.5–16.3)	17.4	(17.1–17.6)	17.5	(17.2–17.7)
Dyspnoea, CR-10	6.2***	(5.8–6.6)	7.9	(7.6–8.3)	8.0	(7.7–8.4)

ISWT: incremental shuttle walking test, ICT: incremental cycle test, CPET: semi steady-state cardiopulmonary exercise test, SpO_2_: oxygen saturation in blood measured by pulsoximeter, RPE: Borg scale for ratings of perceived exertion, CR-10: Borg scale for dyspnoea, VO_2 _peak: peak oxygen uptake, ***: p < 0.0001 compared to ICT and CPET, ^†: ^p < 0.05 compared to ICT. Mean (95% CI).

Fifty-two subjects repeated the ISWT within a week. The difference between the two tests was not significant for any variable measured, mean difference in walking distance being 9 ± 38 m or 3% ± 12% (p = 0.09). All calculations were therefore based on the first ISWT. This subgroup did not differ from the larger group in any baseline characteristics.

### Correlations and estimated W peak

**Figure 1:**
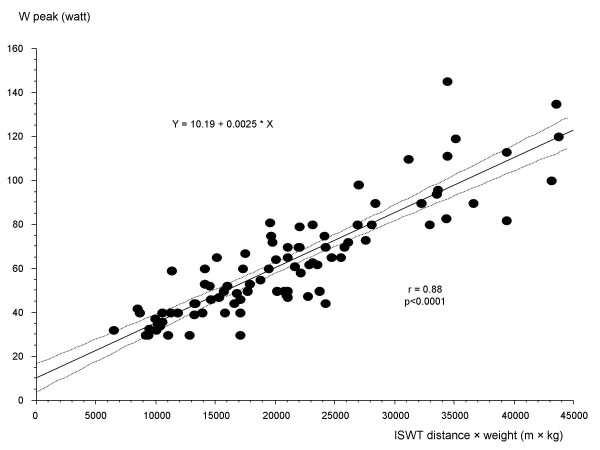
The correlation between the peak exercise capacity (W peak) measured by an incremental cycle ergometer test and the distance walked (m) on an incremental shuttle walking test (ISWT) multiplied by body weight (kg). Regression line and 95% confidence bands. n = 93

**Figure 2:**
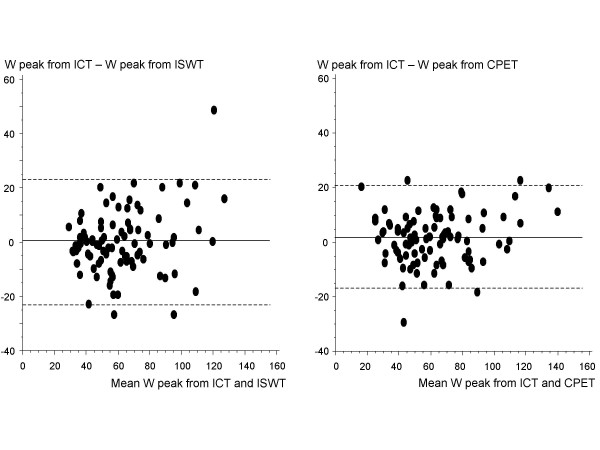
**Scatterplots (Bland-Altman)**. On the left, the difference between measured maximum exercise capacity (W peak) measured by incremental cycle test (ICT) and estimated W peak from performance on incremental shuttle walking test (ISWT) against the mean values of those two. On the right, the difference between measured W peak from ICT and estimated W peak from measured maximum oxygen uptake on semi steady-state cardiopulmonary exercise test (CPET) [16] against the mean of those two. Whole line: mean difference, dotted lines: ± 2SD.

There was a significant correlation (r = 0.88, p < 0.0001) between ISWT distance × body weight and the measured W peak from the incremental cycle test: W peak = 0.0025 × distance (m) × body weight (kg) + 10.19 (Fig. 1). W peak estimated from performance on ISWT by this equation was 62 (57–66) W and did not differ significantly from the measured W peak (62 (57–68) W, p = 0.7). For comparison, W peak was estimated from the measured VO_2 _peak by the equation derived from Åstrand [16], resulting in an estimated W peak of 61 (56–66) W. This was not significantly different from the measured W peak or the estimated W peak from ISWT above. A strong agreement was found between the measured and estimated values of W peak with a minor tendency to overestimation at the lower range and underestimation at the higher range of performance both when estimated from ISWT and from VO_2 _peak (Fig. 2). There is one outlier where ISWT clearly underestimated W peak (Fig. 2). This was the only subject who would have been able to run at the end of the ISWT, which, however, is not allowed in a walk test.

No significant differences were found between women and men regarding the relationship between the different exercise tests.

### Comparisons with estimation based on previous findings

To test whether W peak could be estimated by use of the equation by Singh et al [5], we estimated VO_2 _peak from their equation and then converted this estimated VO_2 _to estimated W peak (as above) [16]. Using the equation by Singh et al, VO_2 _peak was underestimated compared to our measurements (-171 ± 58 ml/min or -16 ± 15 %, p < 0.0001) which consequently resulted in an underestimation of W peak (-14 ± 13 W, p < 0.0001).

## Discussion

In the present study, ISWT distance × body weight was a good predictor of W peak measured by an incremental cycle test in patients with moderate or severe COPD. The fact that W peak estimated from ISWT was as accurate as W peak estimated from VO_2 _is of clinical importance, as the ISWT is much simpler and cheaper than a laboratory cycle test.

Our results confirm the findings of others that there is an excellent correlation between performance on ISWT and laboratory testing [5,6,24]. Although the correlation in our study between VO_2 _and distance walked was almost identical with the findings of Singh et al [5], applying their equation in our material resulted in a significant underestimation. One likely explanation to this could be the difference in the number of subjects, as in their study 19 subjects were included, compared to 93 subjects in the current study. As Singh et al performed two ISWT, and used the second test in their analysis, that might explain some of the inconsistency between their equation and ours, even though no significant improvement on retesting was found in our material. The majority of patients in the study by Singh et al were men while our study was dominated by women. Gender did not significantly affect the relationship between the different tests in our analysis, but this might need further investigation, as only 26% of our subjects were men. The fact that Singh et al compared two walking tests whereas we compared walking and cycling might also explain the difference to some extent, as there is a known difference in metabolic adaptations during walking and cycling [8,15]. However, as VO_2 _peak measured on a treadmill test has been found to be higher than [8] or equal to [25] VO_2 _peak measured on a cycle test in patients with COPD, a regression equation derived from a treadmill test could be expected to overestimate VO_2 _peak on a cycle rather than the opposite.

All patients who were referred to pulmonary rehabilitation and fulfilled the inclusion criteria during the time of the study agreed to participate. Therefore, we conclude that the study is representative of a COPD-population referred to rehabilitation including physical training. The wide range of lung function impairment and age of the participants also enhances the relevance of our sample. In Sweden, women have caught up with men in the prevalence of and mortality in COPD [26]. The majority of women referred to pulmonary rehabilitation during the time of the study is noticeable and we can only speculate that this might indicate that women were either more often offered referral to pulmonary rehabilitation by their physicians or were more likely to accept such an offer than the men.

Heart rate, RPE- and CR-10-scores were significantly lower in ISWT than in the cycle tests. The cycle tests were mainly limited by breathlessness and/or exertion whereas the ISWT was, in most cases, limited by the incapability to increase the speed of walking. During walking it is difficult to increase walking speed above a certain level. Some treadmill test protocols are therefore constructed to increase inclination rather than speed [27].

SpO_2 _decreased more by walking than cycling in the current study, quite in line with previous findings [8,14,24]. It has been speculated that the positional differences between walking and cycling could lead to less effective breathing during walking and thus more desaturation [15]. In spite of the above differences between walking and cycling, our results demonstrated that in patients with moderate to severe COPD it was possible to estimate cycle performance from an ISWT quite as accurately as when estimating performance from one laboratory cycle test to the other. As equations work both ways, our findings also make it possible to estimate distance walked (and thereby walking speed) on ISWT from W peak. This could be useful in clinics where laboratory cycle testing is routine practice but exercise training prescription is mainly walking. As the outlier in our study illustrates, the ISWT can be expected to mimic performance in a symptom-limited cycle test only in subjects that reach their exercise limit by brisk walk, i.e. are unable to run, as otherwise the subjects would not be close to their peak capacity during the test. Being able to run is, however, a very rare condition in COPD-patients referred to rehabilitation, implying that this does not undermine the use of ISWT in patients with COPD.

We used the first ISWT (no training test) for our analyses. Due to the patient's poor condition or because of time constraints it is often not feasible to perform a training test in clinical practice. Thus, it was clinically relevant to present our calculations based on the first ISWT. This was also supported by the finding that the walking distance did not increase at the second test performed within a week. Control calculations were done by using results (not shown) from the second ISWT (n = 52) in our material and no differences were found. Significant difference in walking distance has previously been found on repeated testing with ISWT in patients with COPD [4,28]. It is not clear why our results are inconsistent with previous studies regarding the repeatability of ISWT, but, as noted above, the number of subjects and gender distribution is somewhat different from the other studies, and this might affect the repeatability.

It could be argued that the cycle tests should have been performed in random order. The present design was chosen because it enabled us to adjust the progressive pattern of the CPET to keep exercise time within reasonable limits when W peak from the incremental cycle test was known. Although all subjects rested between the tests, we can not rule out the possibility that the order of the tests could have influenced the results. However, as peak heart rate, CR-10 scores and ratings of perceived exertion (RPE) were almost identical in the two cycle tests, and W peak could be estimated from VO2 peak as expected [16], we consider this error as relatively small.

Being able to estimate W peak by ISWT for clinical purposes does not make ISWT a perfect substitute for the incremental cycle test or other forms of laboratory tests. There is, of course, some variation between estimated and measured peak values on an individual basis, but most importantly the safety aspect of the laboratory tests is beyond the ISWT. Therefore, for COPD patients considered at increased risk for cardiovascular hazards, patients that have never had proper cardiologic assessment or patients with uncertain diagnosis, laboratory tests must be considered as the first choice. However, for COPD-patients of minor risk of cardiovascular incidents, and where laboratory tests are scarce, the ISWT could be used as an alternative for estimating W peak.

## Conclusion

Peak exercise capacity measured by an incremental cycle test could be estimated from an ISWT with similar accuracy as when estimated from peak oxygen uptake.

## Competing interests

The author(s) declare that they have no competing interests.

## Authors' contributions

All authors participated in the design of the study and writing the manuscript as well as approving the final manuscript. HH and RHA participated in data collection, RHA drafted the manuscript.
